# Identification and Comparative Analysis of Premature Senescence Leaf Mutants in Rice (*Oryza sativa* L.)

**DOI:** 10.3390/ijms19010140

**Published:** 2018-01-03

**Authors:** Yan He, Liangjian Li, Zhihong Zhang, Jian-Li Wu

**Affiliations:** State Key Laboratory of Rice Biology/Chinese National Center for Rice Improvement, China National Rice Research Institute, Hangzhou 310006, China; chinayanyan@163.com (Y.H.), 15958018630@163.com (L.L.), 13650950747@163.com (Z.Z.)

**Keywords:** premature senescence, chlorophyll, abscisic acid, reactive oxygen species, ultrastructure, rice (*Oryza sativa* L.)

## Abstract

Premature leaf senescence negatively impacts the grain yield in the important monocot rice (*Oryza sativa* L.); to understand the molecular mechanism we carried out a screen for mutants with premature senescence leaves in a mutant bank generated by ethyl methane sulfonate (EMS) mutagenesis of elite *indica* rice ZhongJian100. Five premature senescence leaf (*psl15*, *psl50*, *psl89*, *psl117* and *psl270*) mutants were identified with distinct yellowish phenotypes on leaves starting from the tillering stage to final maturation. Moreover, these mutants exhibited significantly increased malonaldehyde content, decreased chlorophyll content, reduced numbers of chloroplast and grana thylakoid, altered photosynthetic ability and expression of photosynthesis-related genes. Furthermore, the expression of senescence-related indicator *OsI57* was significantly up-regulated in four mutants. Histochemical analysis indicated that cell death and reactive oxygen species (ROS) accumulation occurred in the mutants with altered activities of ROS scavenging enzymes. Both darkness and abscisic acid (ABA) treatments could induce leaf senescence and resulted in up- or down-regulation of ABA metabolism-related genes in the mutants. Genetic analysis indicated that all the premature senescence leaf mutants were controlled by single non-allelic recessive genes. The data suggested that mechanisms underlying premature leaf senescence are likely different among the mutants. The present study would facilitate us to further fine mapping, cloning and functional characterization of the corresponding genes mediating the premature leaf senescence in rice.

## 1. Introduction

Leaf senescence is the final stage of leaf development, in which intracellular organelles and macromolecules are actively destabilized to relocate nutrients into developing tissues or storage organs [1]. The timing of leaf senescence generates ultimately a great impact on the total biomass production. Understanding the process of leaf senescence caused either by environmental stresses or internal genetic factors is extremely important for the breeding of higher-yielding crops with optimized nutritional qualities [2].

Chlorophyll plays a central role in photosynthesis by forming complexes with thylakoid-membrane proteins such as Photosystem I (PSI), Photosystem II (PSII), and the cytochrome *b6f* complex [3]. The most striking feature of premature senescence leaf mutants is the leaf yellowish phenotype due to the breakdown of chlorophyll during chloroplast degeneration and hydrolysis of macromolecules such as proteins and nucleic acids, which finally results in mitochondria and nuclei dissociation and cell death [4,5]. For example, rice *pse(t)* mutant displays brown spots and yellowish color on the upper leaves which ultimately wilt [6]. Similarly, *psd128* exhibits yellowish with brown spot leaves at the 6-leaf stage and the plants die at the heading stage [7]. It has been shown that chlorophylls and proteins in leaves are largely degraded during the process of rice plant senescence [7,8,9]. Structurally, the thylakoid membranes are damaged, the number of grana decreases in chloroplasts while the number of osmiophilic granules of chloroplast increases during the senescence process, leading to the damage and degradation of chloroplasts as well as the final decline and loss of photosynthesis [7,10].

Reactive oxygen species (ROS) either from extracellular or intracellular sources plays a fundamental role during leaf senescence. Over accumulation of ROS, including O_2_^−^ and H_2_O_2_, causes leaf senescence, even cell death resulting from the impaired structure and function of chloroplasts and mitochondria [11]. ROS scavenging systems such as peroxidase (POD), superoxide dismutase (SOD) and catalase (CAT) are largely disturbed in senescence rice plants [12]. In addition, senescence plants also exhibit a high level of malonaldehyde (MDA), an indicator of cellular membrane damage due to lipid peroxidation [7,13]. External stimuli like darkness and endogenous signals such as abscisic acid (ABA) can promote leaf senescence of rice in advance [14,15]. ABA is a typical plant hormone with a variety of functions during biotic stresses, abiotic stresses and leaf senescence [16]. It has been revealed that ROS signals, especially H_2_O_2_, are involved in ABA-induced rice leaf senescence [17].

To date, as many as 132 rice senescence-associated genes (SAGs) distributing on all the 12 chromosomes have been annotated in the Leaf Senescence Database (http://psd.cbi.pku.edu.cn/). These SAGs are divided into the following five groups: (I) natural senescence, (II) dark induced senescence, (III) nutrition deficiency induced senescence, (IV) stress induced senescence and (V) others [18]. Leaf senescence is a highly coordinated process regulated by a large number of SAGs, which are upregulated during senescence [19]. The corresponding mutants of SAGs generally can be grouped into two major categories according to their phenotypes: premature senescence mutants and delayed senescence mutants [20]. Natural variants or mutants that exhibit delayed senescence are generally called “stay-green” [21], such as *nyc1* [10], *nyc3* [3] *sgr* [22] and *nol* [23] mutants of rice. There are much more premature senescence mutants reported so far compared with the delayed senescence mutants in rice, for instance, the *ospse1* [20], *psd128* [7], *es1-1* [24], *lts* [25], *rls1* [9], *ps1-D* [5] and *noe1* [26] mutants, which are involved in different complex regulatory networks of senescence.

In this study, we identified five premature leaf senescence mutants (*psl15*, *psl50*, *psl89*, *psl117* and *psl270*) from an ethyl methane sulfonate (EMS) mutant bank of rice cultivar ZhongJian100. We further characterized their performance on agronomic traits, their physio-biochemical properties including chlorophyll contents, chloroplast structure, photosynthetic ability, response to darkness and ABA, expression profile of ABA and senescence-related genes and the genetic controls of their premature senescence phenotypes. Our results would provide the basis for the isolation of these premature senescence genes and the elucidation of the senescence mechanism in rice.

## 2. Results

### 2.1. Phenotype of Premature Senescence Leaf Mutants

**Figure 1:**
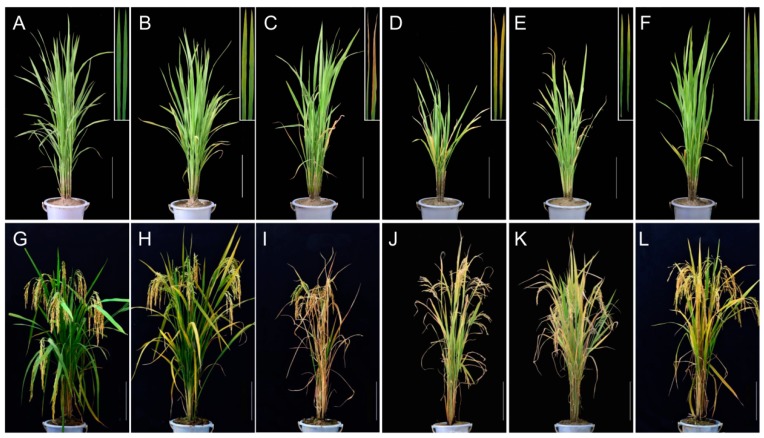
Phenotypes of psl mutants and wild type (WT). (**A**) WT at the tillering stage; (**B**) *psl15* at the tillering stage; (**C**) *psl50* at the tillering stage; (**D**) *psl89* at the tillering stage; (**E**) *psl117* at the tillering stage; (**F**) *psl270* at the tillering stage; (**G**) WT at the grain-filling stage; (**H**) *psl15* at the grain-filling stage; (**I**) *psl50* at the grain-filling stage; (**J**) *psl89* at the grain-filling stage; (**K**) *psl117* at the grain-filling stage; (**L**) *psl270* at the grain-filling stage. Insets show magnified views of bottom second leaves. Scale bar = 20 cm.

Under field and greenhouse conditions in Hangzhou, China, *psl89* and *psl117* displayed distinct yellowish leaf phenotypes around 45 days after sowing. Moreover, obvious retarded growth occurred both in *psl89* and *psl117* mutants compared with wild type (WT) (Figure 1D,E). On the other hand, both *psl15* and *psl270* showed only a slightly yellowing leaf phenotype on the older leaves about 60 days after sowing till to the end of tillering stage (Figure 1B,F). Approximately 65 days after sowing, *psl50* exhibited a brown and wilted phenotype on the bottom older leaves, which become more severe at the maximum tillering stage (Figure 1C). With the development of the plants, the mutants displayed a very significant premature senescence phenotype with deep-yellowing and wilted leaves at the heading and grain-filling stage compared with WT (Figure 1G–L). It was worth noting that *psl50* exhibited a rapid leaf aging and death at the grain-filling stage (Figure 1I), and consequently led to a significant loss of grain yield (Figure S1B,C). Performance of agronomic traits including the number of panicles per plant, length of panicle, seed-setting rate and 1000-grain weight were remarkably reduced in the mutants (Table 1). The differences of plant height between *psl89*, *psl117*, *psl270*, and WT were attributed to the differences of their internode lengths as well as the panicle lengths (Table 1 and Figure S1A). Furthermore, the heading dates of *psl89* and *psl117* mutants were approximately 10 days longer than those of the other three mutants and WT.

### 2.2. Alterations of Photosynthetic and Senescence-related Parameters

**Figure 2:**
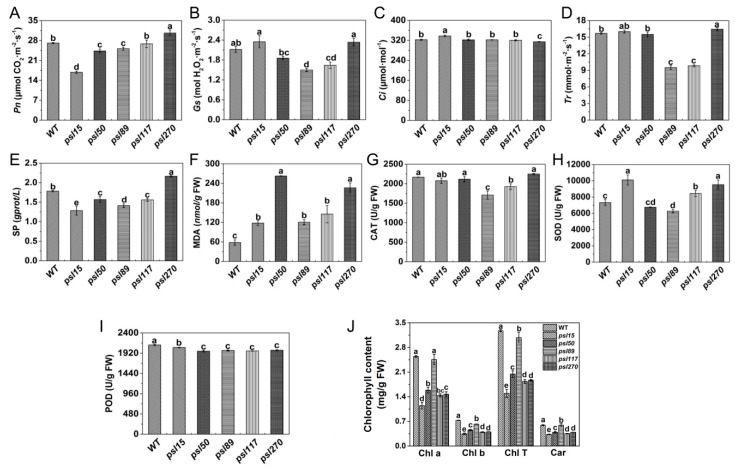
Photosynthetic and senescence-related parameters of WT and mutants. (**A**) Net photosynthetic rate (*Pn*); (**B**) stomatal conductance (*Gs*); (**C**) transpiration rate (*Tr*); (**D**) intercellular CO_2_ concentration (*Ci*); (**E**) soluble proteins (SP) content; (**F**) malonaldehyde (MDA) content. (**G**) Catalase (CAT) activity; (**H**) superoxide dismutase (SOD) activity; (**I**) peroxidase (POD) activity; (**J**) Contents of photosynthetic pigments. Chl a, chlorophyll *a*; Chl b, chlorophyll *b*; Chl T, total chlorophyll; Car, carotenoid. All experiments were carried out using flag leaves at the heading stage. Error bars indicate Mean ± SD (*n* = 3). Different letters indicate a statistical difference at *p* ≤ 0.05 by Duncan’s test.

To investigate the influences of premature senescence leaves on photosynthesis efficiency, we examined the photosynthetic parameters of the flag leaves at the heading stage. As an important photosynthetic parameter, net photosynthetic rate (*Pn*) affects accumulation of dry matter of crops to some extent. We found that the value of *Pn* was similar between *psl117* and WT, and *Pn* values of *psl15*, *psl50* and *psl89* were significantly lower than that of WT, in contrast, *Pn* value of *psl270* was significantly higher than that of WT (Figure 2A). The lower level of *Pn* could cause the accumulation of intercellular CO_2_, thus the intercellular CO_2_ concentration (*Ci*) in *psl15* mutant was significantly higher than those of WT and the other four mutants (Figure 2C). In contrast, the higher level of *Pn* in *psl270* might consume more CO_2_, leading to a significant decrease of *Ci* in *psl270* (Figure 2A,C). There were general positive correlation between the values of stomatal conductance (*Gs*) and transpiration rate (*Tr*) [27]. Hence, it is reasonable that *psl270* exhibited the highest levels of *Gs* and *Tr* while *psl89* and *psl117* showed the lowest level of *Gs* and *Tr* (Figure 2B,D).

At the heading stage, we also examined the senescence-related parameters, including the content of soluble protein (SP) and malonaldehyde (MDA), and the enzymatic activities of ROS scavenging systems including catalase (CAT), superoxide dismutase (SOD), and peroxidase (POD). Our results showed that the level of SP in *psl270* was significantly higher than that of WT, while the contents of SP in the other mutants were apparently lower than that of WT (Figure 2E). This might also explain why *psl270* has the highest level of *Pn* among the mutants. In comparison with WT, the MDA contents of all five mutants were significantly increased at the heading stage (Figure 2F), consistent with the presence of early senescence phenotype in the mutants. The activities of CAT were significantly lower in *psl89* and *psl117* than that of WT, while CAT activities were similar to WT in *psl15*, *psl50* and *psl270* (Figure 2G). The activities of SOD in *psl15*, *psl117*, and *psl270* were apparently higher than that of WT while the SOD activity was much lower in *psl89* than that of WT (Figure 2H). The activities of POD in all the mutants were significantly lower than that of WT (Figure 2I). In terms of mutants, the activities of all three enzymes were significantly decreased in *psl89*, indicating that *psl89* might have lost the ability for detoxification of ROS. Besides the role in detoxification of ROS, POD is also closely related to the lignification of plant tissues and has a lower activity in the aging plant tissues [28]. This may explain why all five mutants displayed the lower level of POD activities.

To explore the direct cause for the yellowish leaf phenotype of the mutants, we examined photosynthesis-related pigment contents at the heading stage. The results indicated that the pigment contents in the flag-leaves of the mutants including chlorophyll *b* (Chl *b*), total chlorophyll (Chl T) and carotenoid (Car) were significantly lower than those of WT (Figure 2J). In fact, significant differences of chlorophyll contents in five mutants had appeared at the seedling stage (Figure S1D), even though there were no visual differences in phenotype among these five mutants and WT at this stage. Taken together, all the results above indicated that the five mutants did possess typical premature senescence characteristics mainly with yellowish leaves, elevated MDA contents, and decreased photosynthetic pigment levels and ability.

### 2.3. Impaired Chloroplast Development in the Mutants

**Figure 3:**
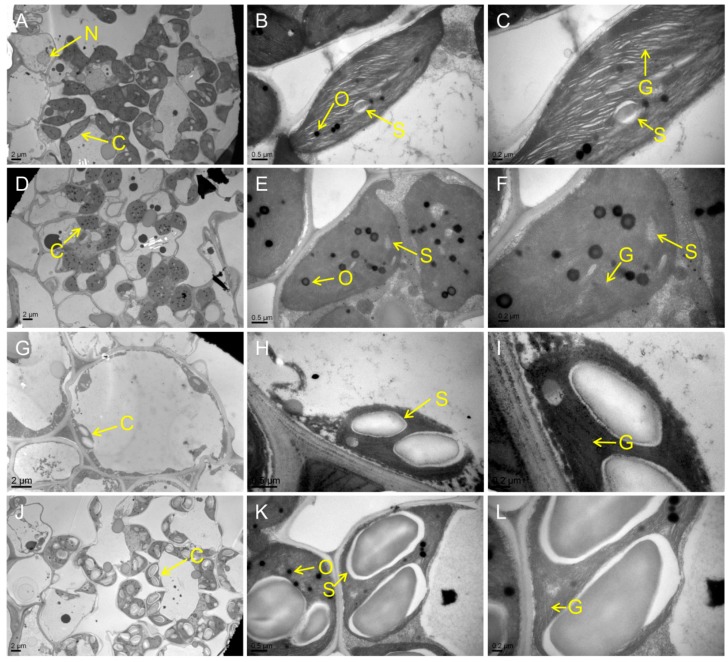

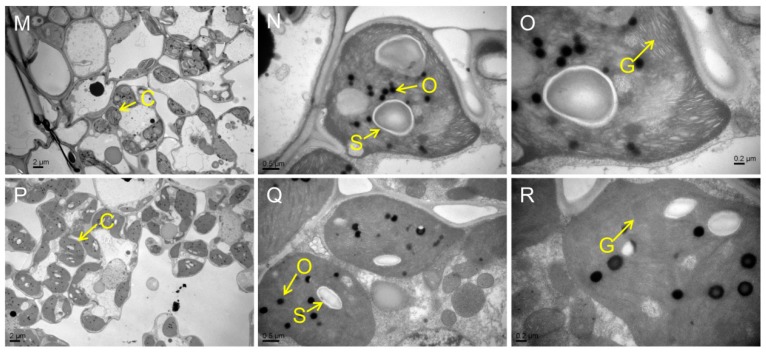
Ultrastructure of chloroplast in the mutants and WT at the tillering stage. (**A**–**C**) WT. (**D**–**F**) *psl15*. (**G**–**I**) *psl50*. (**J**–**L**) *psl89*. (**M**–**O**) *psl117*. (**P**–**R**) *psl270* mutant. C, chloroplast; N, nucleus; O, osmiophilic granule; S, starch granule; G, grana thylakoid.

Chloroplast degradation is one of the cellular characteristics of leaf senescence in rice. To explore the chloroplast development in the mutants, we compared the ultrastructure of chloroplast in WT and the mutants at the tillering stage by transmission electron microscopy (TEM). The results showed that the number of chloroplast and grana thylakoid were lower in all mutants than those of WT (Figure 3A–R). Among them, the chloroplast structure was degraded most seriously in *psl50* mutant (Figure 3G–I). As for *psl89* mutant, the number of starch granules was increased and the size of starch granules was much larger compared with WT (Figure 3J–O). Furthermore, the number of osmiophilic granules increased in the chloroplasts of *psl15*, *psl117*, and *psl270* mutants compared with WT. All these results demonstrated that the EMS-induced mutations resulted in chloroplast dysplasia in all mutants.

### 2.4. Premature Senescence Leaf phenotypes Are Controlled by Single Recessive Genes

All F_1_ plants from the crosses of *psl15*/ZJ100, *psl50*/ZJ100, *psl89*/IR64, *psl117*/ZJ100 and *psl270*/80A90YR72 showed a normal green phenotype similar to WT. The number of WT plants and the mutant-type F_2_ individual plants fitted to the expected 3:1 ratio in the corresponding five F_2_ populations (Table 2). These results indicated that the premature senescence leaf phenotypes in all five mutants were controlled by single recessive genes.

To determine whether these five mutants are allelic to each other, we further intercrossed the five mutants with each other and investigated the phenotypes of F_1_ plants. The results showed that each of the mutant displayed a distinct phenotype and is non-allelic to each other except the allelism between *psl15* × *psl89* and *psl50* × *psl117* could not be determined because their F_1_ plants from these two crosses failed to survive (Table 3 and Figure S2)*.*

### 2.5. Darkness and ABA Induce Leaf Senescence

**Figure 4:**
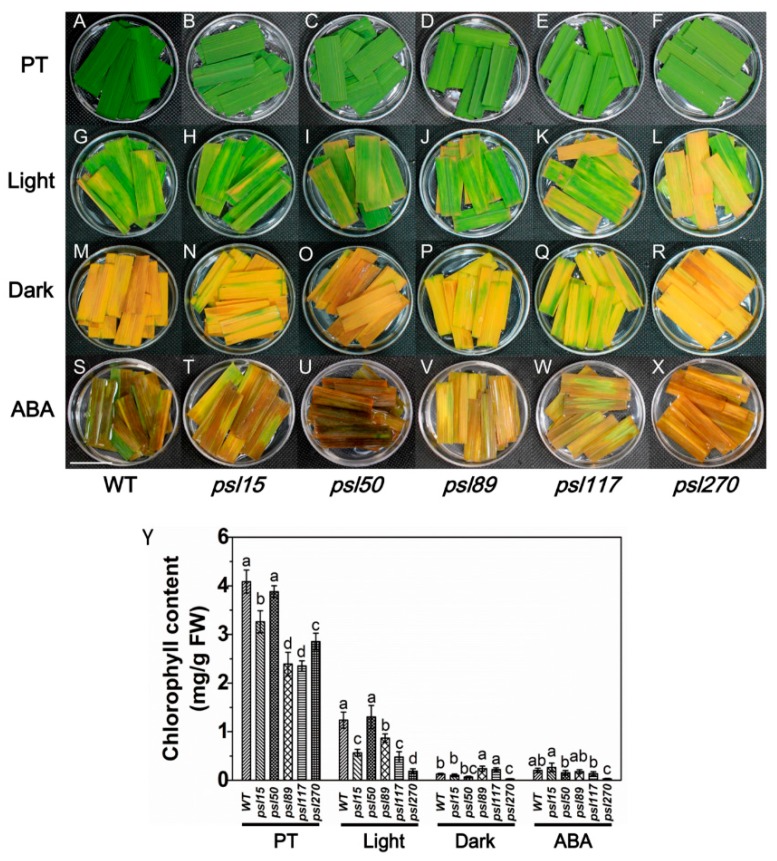
Darkness- and abscisic acid (ABA)-induced leaf senescence. (**A**–**X**) Detached top second leaves from the WT and five mutants at the tillering stage were incubated with continuous light (H_2_O), darkness (H_2_O), and 200 μM ABA (continuous light) at 28 °C for 5 d. PT, Pretreatment. Scale bar = 2 cm. (**Y**) Chlorophyll content of the detached top second leaves at tillering stage. Error bars indicate means ± SD (*n* = 3). Different letters indicate a statistical difference at *p* ≤ 0.05 by Duncan’s test.

Darkness is one of the most powerful known external stimuli of leaf senescence [4]. Consequently, it is used frequently as an effective method to induce synchronous senescence [29,30]. To confirm whether leaf senescence progress could be accelerated in darkness, the detached flag leaves from the mutants were incubated in darkness and control light conditions for five days. The results showed that *psl15*, *psl89*, *psl117* and *psl270* had lower chlorophyll contents than that of WT while *psl50* had a similar level of chlorophyll to WT before treatment (Figure 4A–F,Y). The chlorophyll levels in detached leaves changed in a similar pattern after 5 d treatment in continuous light although *psl270* showed the most rapid decline in chlorophyll level with the most prominent yellowish phenotype (Figure 4G–L,Y). Under darkness conditions, although all the mutants and WT displayed darkness-induced yellowing phenotype, and the detached leaves of *psl89* and *psl117* exhibited relatively delayed senescence with greener leaves and higher chlorophyll contents compared with the other mutants and WT (Figure 4M–R,Y). Again, *psl270* displayed apparently accelerated senescence and significantly reduced chlorophyll content compared with the remaining mutants and WT in darkness (Figure 4M–R,Y).

Leaf senescence is a genetically controlled developmental process that can be modulated by a variety of phytohormones, especially ABA, that plays a critical role in leaf senescence [31,32]. We thus further examined the senescence symptoms of detached leaves after 5 d ABA treatment. The results indicated that significantly accelerated senescence under ABA treatment was observed in all materials tested compared with that under light conditions (Figure 4G–L,S–X). Interestingly, only *psl270* showed significantly reduced chlorophyll content under ABA treatment (Figure 4Y). Taken together, our results revealed that *psl270* was likely more sensitive both to the stimuli of darkness and ABA than WT and the other mutants.

### 2.6. ROS Accumulation, Cell Death and DNA Fragmentation Occur in the Mutants

**Figure 5:**
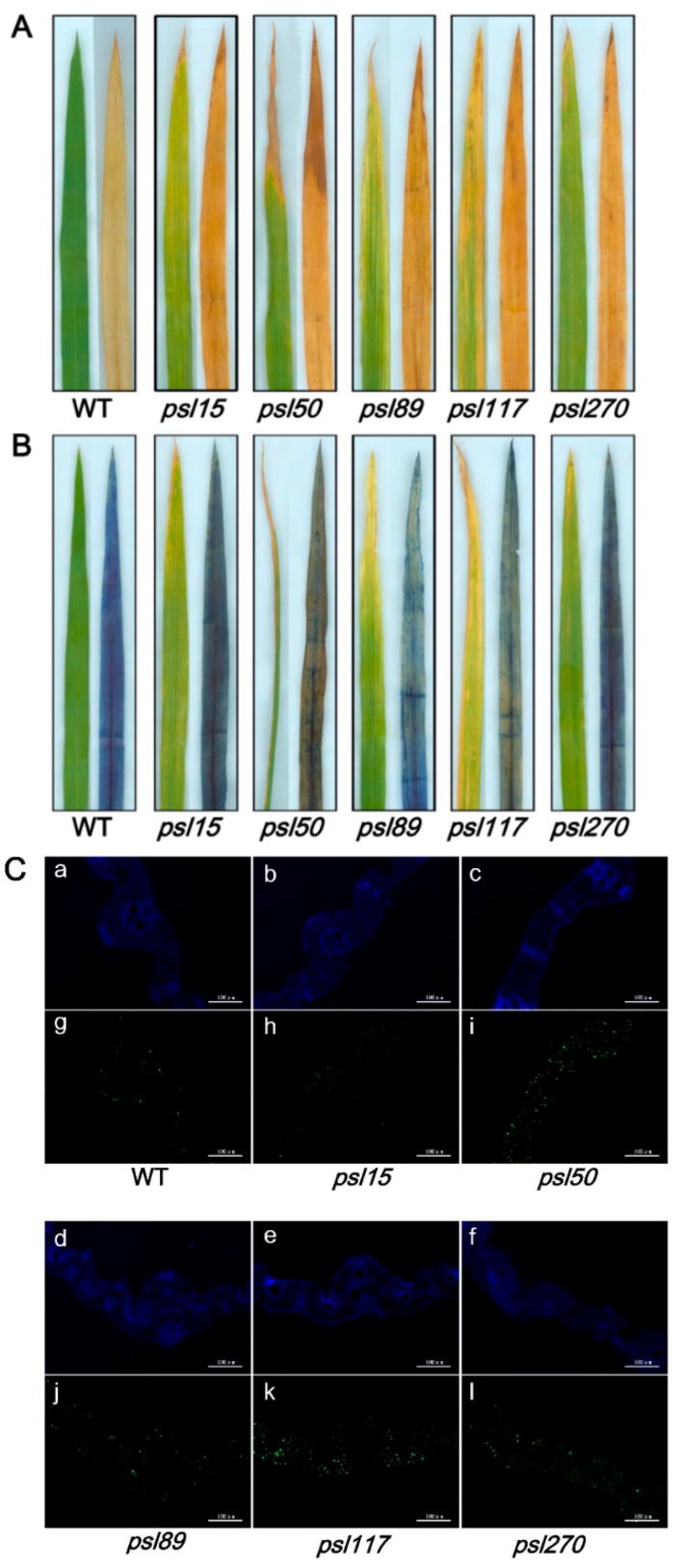
Histochemical analysis and terminal deoxyribonucleotidyl transferase-mediated dUTP nick-end labeling (TUNEL) assays in WT and the mutants. (**A**) 3,3-diaminobenzidine (DAB) assay at the tillering stage; (**B**) Trypan Blue staining at the tillering stage; (**C**) TUNEL assay at the tillering stage. Blue signal represents 4′,6-diamino-phenylindole (DAPI) staining; green color represents positive result. a–f are negative controls for g–l, respectively. The scale bars indicate 100 μM.

To detect whether accumulation of hydrogen peroxide (H_2_O_2_) and cell death occur in the mutants, the leaf samples at the tillering stage from WT and the mutants were stained with 3,3′-diaminobenzidine (DAB) and Trypan Blue, respectively. The results showed that increased level of brown precipitations were observed in the mutants compared with WT, indicating that the presence of H_2_O_2_ accumulation did occur in the mutants (Figure 5A). Similarly, more number of blue spots was detected in the mutant leaves especially in *psl50*, *psl89*, *psl117* and *psl270* after Trypan Blue staining, indicating that the presence of cell death also occurred in these four mutants on the progression of premature leaf senescence (Figure 5B).

To confirm the cell death in the mutants at cellular and molecular levels, we further performed a terminal deoxyribonucleotidyl transferase-mediated dUTP nick-end labeling (TUNEL) assay on the bottom second leaves at the tillering stage. The same leaf sections of bottom second leaves were simultaneously stained with 4′,6-diamino-phenylindole (DAPI) to reveal the nuclei (blue). The results showed that a few number of nucleus (green) were TUNEL positive in WT, whereas numerous nuclei were TUNEL positive in leaf sections of *psl50*, *psl89*, *psl117* and *psl270* mutants (Figure 5C). These results indicated that the mutations induced DNA damage especially in *psl50*, *psl89*, *psl117* and *psl270*, corresponding to cell death detected in Trypan blue staining (Figure 5B).

### 2.7. Alterations of ABA Contents and ABA-Related Gene Expression

**Figure 6:**
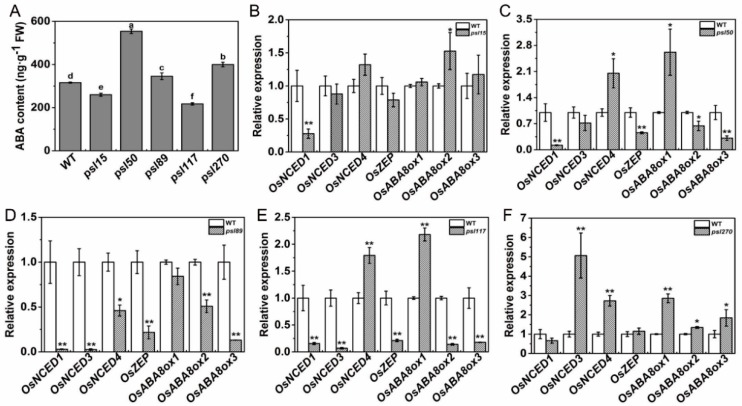
ABA contents and expression of ABA-related genes at the heading stage. (**A**) ABA contents in the flag leaves; (**B**–**F**) expression of ABA-related genes including biosynthetic genes *OsNCED1* (*LOC_Os02g47510*), *OsNCED3* (*LOC_Os03g44380*), *OsNCED4* (*LOC_Os07g05940*) and *OsZEP* (*LOC_Os04g37619*) and inactivation genes *OsABA8ox1* (*LOC_Os02g47470*), *OsABA8ox2* (*LOC_Os08g36860*) and *OsABA8ox3* (*LOC_Os09g28390*). Error bars indicate means ± SD (*n* = 3). * *p* ≤ 0.05, ** *p* ≤ 0.01; Student *t* test. Different letters indicate a statistical difference at *p* ≤ 0.05 by Duncan’s test.

We have shown that external ABA treatment causes senescence in all the mutants as well as WT (Figure 4Y). To further investigate whether the premature leaf senescence was associated with the internal ABA levels in the mutants, we measured the ABA contents of flag leaves at the heading stage. The results showed that the contents of ABA in *psl15* and *psl117* were apparently lower than that of WT while the ABA contents in *psl50*, *psl89* and *psl270* were significantly higher than that of WT (Figure 6A). Further examination revealed that the transcription level of the ABA biosynthetic gene *OsNCED1* was significantly down-regulated and the ABA-inactivation gene *OsABA8ox2* was significantly up-regulated in *psl15* mutant (Figure 6B). The expressions of six genes including four ABA biosynthetic genes *OsNCED1*, *OsNCED3*, *OsNCED4*, and *OsZEP* as well as two ABA-inactivation genes *OsABA8ox2* and *OsABA8ox3*, were significantly down-regulated in *psl89* mutant (Figure 6D). The expression patterns of ABA-related genes were similar in *psl50* and *psl117* with a couple of genes (*OsNCED4* and *OsABA8ox1*) were upregulated and 4–5 genes (*OsNCED1*, *OsNCED3*, *OsZEP*, *OsABAox2* and/or *OsABAox3*) were down-regulated (Figure 6C,E) although the ABA contents of these two mutants displayed hugely different from each other compared with WT (Figure 6A). In contrast to *psl89*, the expression levels of five ABA-related genes (*OsNECD3*, *OsNECD4*, *OsABAox1*, and *OsABAox2* and *OsABAox3*) were all significantly upregulated in *psl270* compared with WT (Figure 6F). Our results demonstrated that ABA participated in the mediation of premature leaf senescence probably in different manners in the mutants.

### 2.8. Differential Expression of Genes Associated with Senescence, Chlorophyll Metabolism and Photosynthesis

**Figure 7:**
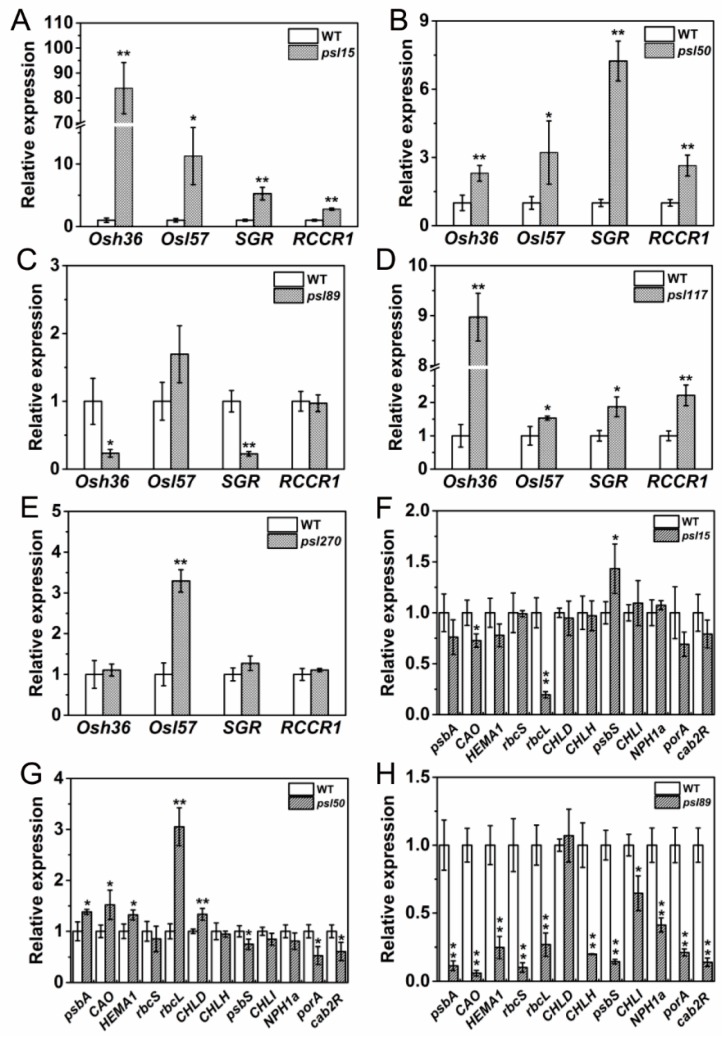

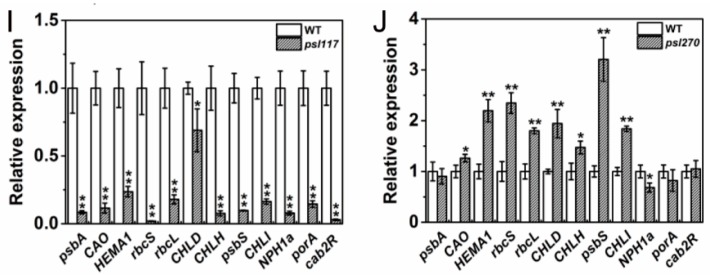
Expression profile of senescence, chlorophyll and photosynthesis-related genes at heading stage. (**A**–**E**) Expression of *Osh36*, *OsI57*, *SGR* and *RCCR1* in WT and the mutants; (**F**–**J**) expression of photosynthesis-related genes in WT and the mutants. Error bars indicate ± SD (*n* = 3). * *p* ≤ 0.05, ** *p* ≤ 0.01; Student *t* test.

Besides ABA-related genes, a large number of genes, including senescence-associated genes (SAGs), chlorophyll metabolism-related genes, and photosynthesis-related genes are well known in participation of leaf senescing process. To examine their performance in the mutants, we determined the expression of two SAGs, *Osh36* and *OsI57* [33], two chlorophyll degradation-related genes, *stay-green* (*SGR*) and *red chlorophyll catabolite reductase 1* (*RCCR1*) [22,34], and a set of photosynthesis-related genes [31] in the flag leaves at the heading stage. The results showed that *Osh36*, *OsI57*, *SGR* and *RCCR1* were all highly expressed (*p* ≤ 0.05) in *psl15*, *psl50* and *psl117* compared with WT (Figure 7A,B,D) while only *OsI57* was highly expressed (*p* ≤ 0.05) in *psl270* at the heading stage (Figure 7E). Unexpectedly, the expression levels of *Osh36* and *SGR* were significantly decreased in *psl89* compared with WT (Figure 7C). Nevertheless, the senescence indicator *OsI57* was significantly upregulated in four out of five mutants. Overall, our results confirmed that the expression of senescence and chlorophyll metabolism-related genes have been altered in the premature senescence leaf mutants.

For the expression of photosynthesis-related genes, a set of 12 genes including *psbA*, *CAO*, *HEMA1*, *rbcS*, *rbcL*, *CHLD*, *CHLH*, *psbS*, *CHLI*, *NPH1a*, *porA* and *cab2R* were chosen for analysis. We found that the expression levels of *CAO* and *rbcL* were significantly increased while *psbS* was significantly decreased in *psl15* compared with WT (Figure 7F). The expression levels of *psbA*, *CAO*, *HEMA1*, *rcbL* and *CHLD* were apparently increased while the expression levels of *psbS*, *porA* and *cab2R* were greatly decreased in *psl50* compared with WT (Figure 7G). Interestingly, the expression profile of these genes were similar in *psl89* and *psl117* with a significant down-regulation of 11/12 and 12/12 genes, respectively (Figure 7H,I). Whereas in case of *psl270* mutant, eight out twelve photosynthesis-associated genes were up-regulated, and only one gene *NPH1a* was down regulated in comparison with WT (Figure 7J), consistent with its stronger photosynthetic capacity (Figure 2A). Taken together, the results indicated that altered expression of photosynthesis-associated genes might have contributed to the premature leaf senescence in the mutants.

## 3. Discussion

Senescence process is complicated and still poorly understood although many SAGs have been identified. In this study, we isolated five premature senescence leaf mutants from an EMS-induced mutant library of ZhongJian100. The premature senescence leaf mutants were characterized by yellowish leaves, high MDA levels, and low chlorophyll contents at the heading stage, and this consequently led to the poor performance of their major agronomic traits at maturity. Each mutant shows a distinct phenotype and is genetically controlled by a single recessive gene. *psl15* is non-allelic to *psl50*, *psl117* and *psl270*; *psl50* is non-allelic to *psl15*, *psl89* and *psl270*; *psl89* is non-allelic to *psl50*, *psl117* and *psl270*; *psl117* is non-allelic to *psl15*, *psl89* and *psl270* while *psl270* is non-allelic to the other four mutants. The allelism between *psl15* and *psl89*, *psl50* and *psl117* could not be determined because their F_1_ plants were not viable. The reason for lethality of F_1_ plants from these two crosses is unknown and requires to be further characterized.

The yellowish phenotype is directly associated with loss of chlorophyll contents and impaired development of chloroplasts. This has been well demonstrated in chlorophyll-deficient mutants such as *OscpSRP43* [35]. In the present study, the contents of chlorophyll are significantly decreased in all five mutants at the seedling stage as well as the tillering stage and the heading stage as expected. Decreased chlorophyll contents are associated with impaired development or degradation of chloroplasts in these mutants. Additionally, the mutations in *psl15*, *psl50*, *psl89* and *psl117* mutants also caused negative effects on their photosynthetic capacity, respectively. However, it is worth noting that *psl270* exhibits a better performance on photosynthetic capacity which could be related to its higher content of SP and up-regulation of multiple photosynthesis-related genes compared with WT. It has been shown in a simulation study that lower chlorophyll level is not necessarily a bad trait for plant biomass production [36]. Therefore, even though the elevated photosynthetic capacity and the SP level in *psl270* is rare in premature senescence mutants, while it could be useful in rice breeding for higher yield and better grain quality.

At the molecular level, the senescence process is accompanied with alterations of the expression of photosynthesis-associated genes, SGRs and CDGs. *Osh36* and *OsI57* are two senescence indicators that usually up-regulated in senescence plants. *SGR* encodes a chloroplast protein required to trigger chlorophyll degradation during natural and darkness-induced leaf senescence [22]. *RCCR1* is participated in the breakdown of chlorophyll [37]. In the present study, the up-regulation of *Osh36* and *OsI57* are observed in *psl15*, *psl50*, and *psl117* as expected. A slightly different pattern from these three mutants is that only the expression of *OsI57* is up-regulated in *psl270*, probably indicating that up-regulation of *OsI57* is enough to cause premature senescence phenotype. Unexpectedly, the expression of *Osh36* is in contrast down regulated while *OsI57* expression is not changed in *psl89*, reflecting that the premature senescence of *psl89* might be controlled by a different mechanism. In fact, the expression of *SGR* and *RCCR1* is also up-regulated in *psl15*, *psl50*, and *psl117*, while it is down-regulated in *psl89*, further indicating that a likely different mechanism of premature senescence might have involved in *psl89*. Like rice, the mutation of *Arabidopsis STAY-GREEN* (*SGR*) which encodes Mg-dechelatase would also cause a strong stay-green phenotype because it catalyzes the first step of the chlorophyll degradation pathway [38]. It might indicate that the enzymatic properties of *SGR* remain similar in both of monocots and dicots. Most of the mutants of chlorophyll degradation enzymes, such as PPH [39], PaO [40], and CBR [41], exhibit a stay-green phenotype. Whereas in our study, all the mutants showed premature yellow aging, hence we could conclude that there are not dysfunctions of the chlorophyll degradation enzymes in all five mutants. In addition, both *SGR* and *RCCR1* expressions in *psl270* are similar to WT, thus the decreased chlorophyll level in the mutant might be not associated with the break-down of chlorophyll, and whether it is purely associated with the up-regulation of *OsI57* is yet to be characterized.

ABA might act as an original inducer in initiative of senescence and plays an important role in triggering leaf senescence [42]. ABA anabolic enzyme 9-cis-epoxycarotenoid dioxygenase (NCED) is the key enzyme that controls the synthesis of ABA in rice while the oxidation of ABA to phaseic acid (PA) is catalyzed by ABA catabolic enzyme 8′-hydroxylase which is possibly encoded by three genes (*OsABA8ox1*, *OsABA8-2* and *OsABA8-3*) in rice [43,44]. In the present study, ABA treatment causes senescence in the mutants as well as WT, although the internal ABA levels are significantly different among them. Decreased ABA level in *psl15* can be well explained by the down regulation of ABA biosynthetic gene *OsNECD1* and up-regulation of ABA catabolic gene *OsABAox-2*. Like *psl15*, *psl117* also shows a decreased internal ABA level but more ABA-related genes have been affected including down regulation of five genes (*OsNECD1*, *OsNECD3*, *OsZEP*, *OsABAox-2*, and *OsABAox-3*) and up-regulation of two genes (*OsNECD4* and *OsABAox-1*). It needs to be characterized whether the biosynthetic genes or the catabolic genes contribute more to the lowered internal ABA level. The mutant *psl50* has a similar expression pattern of these genes to *psl117*, but the overall internal ABA level is significantly increased in *psl50*. We figure that unknown genetic factors might have contributed to this difference between the two mutants. It would be necessary to clone the mutation genes and characterize their function in order to explain the differential gene expression patterns. The mutant *psl89* exhibits a significantly increased internal ABA level, but six of these genes except OsABAox-1 are down regulated. Similar to *psl50*, we assume that genes rather than those studied in the present are likely involved in the control of ABA level in *psl89*. In contrast to *psl89*, five genes are all up-regulated in *psl270* that shows a significant increased internal ABA level, a possible explanation is that the rate of ABA synthesis exceeded the rate of ABA catabolism. Overall, ABA level may affect premature senescence of these mutants, but through different mechanisms.

ROS act both as important toxic substances and signaling molecules that play an important role in lipid peroxidation, membrane damage, and consequently in leaf senescence [45]. ROS can be generated by various sources of cellular components [46,47,48,49]. The disturbance in balance between the production and scavenging of ROS may stimulate the formation of membrane lipid peroxidation by regulating the redox status of relevant cellular organelles [50]. In the present study, the activities of three ROS scavenging enzymes SOD, CAT, and POD, are significantly decreased in *psl89*, indicating that this mutant is unable to detoxify ROS which consequently lead to the premature senescence. Similarly, the activities of POD are all apparently decreased in all the mutants, thus POD may contribute to the senescing phenotype. Based on the metal co-factor used by the enzyme, SOD can be classified into three groups of isozyme: copper-zinc SOD (Cu-Zn SOD), manganese SOD (Mn SOD), and iron SOD (Fe SOD), furthermore, the roles of them differ in oxidative stress conditions [51]. An *Arabidopsis* mutant in absence of *Fe-SOD* gene could not produce Fe SOD isozyme but with an increase level of Cu-Zn SOD isozyme, however, the enhanced Cu-Zn SOD activity cannot completely compensate for the deficiency in Fe SOD function [52]. Thus, we speculate that the synergetic balance among these three SOD isozymes might be broken, resulting in changes of activity for each SOD group in *psl15*, *psl117* and *psl270* mutants. Finally, *psl15*, *psl117*, and *psl270* mutants display higher levels of total SOD activities than that of WT. Quantitative determination of internal ROS levels in the mutants and functional analysis of respective genes responsible for the mutations would further help us to clarify the variation and roles of these enzymes. Furthermore, classification of sources of ROS production in each of these mutants would facilitate our understanding of ROS-mediated leaf senescence.

Overall, the results obtained in this study have provided foundations for further studies on the fine mapping, isolation, and functional analysis of corresponding genes governing the premature senescence leaf phenotype.

## 4. Materials and Methods

### 4.1. Plant Materials

Five premature senescence leaf mutants (*psl15*, *psl50*, *psl 89*, *psl117* and *psl270*) with yellowing leaves were obtained from an ethane methyl sulfonate (EMS)-induced *indica* rice ZJ100 mutant bank. These mutants have been selfed for more than 10 generations and the target trait has been stably expressed under the field and greenhouse conditions in Fuyang, Hangzhou, Zhejiang, China, and Lingshui, Hainan, China.

### 4.2. Genetic Analysis and Allelism Tests

Mutants *psl15*, *psl50* and *psl117* were crossed to the wild type ZJ100 (WT), *psl89* was crossed to the parental rice IR64, and *psl270* was crossed to the parental line 80A90YR72, respectively. All F_1_ plants and the F_2_ populations were grown in the paddy field at China National Rice Research Institute (CNRRI) for genetic segregation analysis while the mutants and WT were used for the evaluation of main agronomic traits including plant height, tiller number/plant, panicle length, and 1000-grain weight during the rice growing season in 2016. Means from three replications were used for data analysis. For allelism tests, the mutants were intercrossed among themselves, and the F_1_ plants were grown and phenotyped in the paddy field at CNRRI.

### 4.3. Histochemical Analysis

The premature senescence leaves from the five mutants and WT at the tillering stage were collected for detecting the H_2_O_2_ accumulation by 3,3-diaminobenzidine (DAB) staining and cell death by Trypan blue staining [53], respectively. The pictures were recorded using a HP ScanJet G4010scanner (HP, Shanghai, China).

### 4.4. Measurement of Physio-Biochemical Parameters

Chlorophyll was extracted from 50 mg of fresh flag-leaves. The contents of chlorophyll *a* (Chl *a*), chlorophyll *b* (Chl *b*), total chlorophyll (Chl T) and carotenoid (Car) were determined according to Wellburn [54]. The enzymatic activities of peroxidase (POD), superoxide dismutase (SOD) and catalase (CAT), the contents of malonaldehyde (MDA) and soluble proteins (SP) were measured following the manufacturer’s instructions (Nanjing Jiancheng Bioengineering Institute, Nanjing, China) at the heading stage. Photosynthetic parameters including net photosynthetic rate (*Pn*, μmol·m^−2^·s^−1^), stomatal conductance (*Gs*, mmol·m^−2^·s^−1^), intercellular CO_2_ concentration (*Ci*, μmol·mol^−1^) and transpiration rate (*Tr*, mmol·m^−^^2^·s^−1^) were determined at 9:00–10:00 a.m. under field conditions with a portable L-6400XT (LI-COR, Lincoln, NB, USA). All the measurements were taken at the saturation irradiance with an incident photosynthetic photo flux density (PPFD) of 1200 μmol·m^−2^·s^−1^ and an airflow rate at 500 μmol·s^−1^. Means from three replicates were used for analysis.

### 4.5. TUNEL Assays

The TUNEL assays for DNA fragmentation were performed by using a Fluorescein in Situ Cell Death Detection Kit following the manufacturer’s instructions (Roche, Basel, Switzerland). The methods used for sectioning and fluorescence labeling were described as previously reported [55].

### 4.6. Transmission Electron Microscopy

Leaf sections from mutants and WT at the tillering stage were used to perform transmission electron microscopy according to the method described previously [1]. Samples were stained with uranyl acetate and examined with a Tecnai G2F20 transmission electron microscope at the College of Agriculture and Biotechnology, Zhejiang University.

### 4.7. Darkness and ABA treatment

Fully expanded top second leaves at the tillering stage were excised carefully. The detached leaves were cut into ~2 cm pieces and floated on 20 mL of water or 200 μM ABA solution in Petri dishes. The samples were incubated at 30 °C in darkness and continuous light for five days.

### 4.8. ABA Content Measurement

The flag leaves from WT and the mutants were respectively collected at the heading stage, then immediately frozen in liquid nitrogen, and stored at −80 °C. 100 mg powdered fresh specimen was weighed out and freeze-dried for 3 h, and then extracted with MilliQ water at 4 °C in dark for 16 h. Quantitative analysis of ABA was performed using the Phytodetek ABA enzyme-linked immunosorbent assay (ELISA) Kit (Agdia, Inc., Elkhart, IN, USA) according to the manufacturer’s instructions.

### 4.9. Real-Time Fluorescent Quantitative PCR (qRT-PCR) Analysis

The total RNA was isolated from flag leaves of WT and the mutants at the heading stage using the NucleoZOL Reagent Kit according to the manufacturer’s instructions (MACHEREY-NAGEL, Düren, Germany). The first strand of copy DNA (cDNA) was synthesized using the ReverTra Ace qPCR RT Master Mix with genomic DNA (gDNA) Remover Kit (Toyobo, Osaka, Japan). Real-time fluorescent quantitative PCR was carried out using the FastStar Essential DNA Green Master Kit (Roche, Basel, Switzerland) and performed on a Thermal Cycle Dice^®^ Real Time System (Takara, Kusatsu, Japan). All target genes were normalized to the rice internal control gene *Ubiquitin* (*LOC_Os03g13170*) to detect the relative expression levels. Three biological repeats were conducted to obtain the final results. The primers for the qRT-PCR are listed in Table S1.

## Figures and Tables

**Table 1:** Agronomic traits of the mutants and wild type.

Material	Plant Height (cm)	Panicle Length (cm)	No. Panicle	1000-Grain Weight (g)	Seed-Setting Rate (%)
ZJ100	101.66 ± 2.89a	26.60 ± 0.53a	14.67 ± 0.58b	22.01 ± 0.10a	85.99 ± 0.86a
*M15*	97.50 ± 1.82ab	24.00 ± 0.50bc	11.00 ± 1.00de	21.48 ± 0.12b	76.33 ± 0.75c
*M50*	97.83 ± 1.76ab	23.73 ± 0.72bc	10.33 ± 0.58e	19.53 ± 0.09d	74.88 ± 0.46c
*M89*	95.17 ± 2.25b	19.63 ± 1.23d	13.33 ± 1.15bc	18.80 ± 0.07e	81.14 ± 2.08b
*M117*	83.33 ± 4.72c	22.33 ± 0.86c	16.67 ± 1.15a	20.84 ± 0.20c	74.27 ± 1.63c
*M270*	93.67 ± 4.62b	24.73 ± 1.46b	12.33 ± 1.15cd	21.74 ± 0.32ab	86.32 ± 2.41a

Different letters after each number indicate a statistical difference at *p* ≤ 0.05 by Duncan’s test.

**Table 2:** Genetic analysis of premature senescence leaf mutants.

Cross	F_1_	No. F_2_ Individual		χ^2^_(3:1)_
		Wild-Type	Mutant-Type	
*psl15*/ZJ100	Normal	292	88	0.69
*psl50*/ZJ100	Normal	495	170	0.11
*psl89*/IR64	Normal	457	137	1.19
*psl117*/ZJ100	Normal	501	154	0.77
*psl270*/80A90YR72	Normal	225	63	1.50

**Table 3:** Allelism test of premature senescence leaf mutants.

Cross (Female Parent × Male Parent)	Phenotype of F_1_ Plants	Allelism
*psl15* × *psl50*	Wild-type	Non-allelic
*psl89* × *psl15*	Non-survival	Not determined
*psl117* × *psl15*	Wild-type	Non-allelic
*psl270* × *psl15*	Wild-type	Non-allelic
*psl50* × *psl89*	Wild-type	Non-allelic
*psl117* × *psl50*	Non-survival	Not determined
*psl270* × *psl50*	Wild-type	Non-allelic
*psl89* × *psl117*	Wild-type	Non-allelic
*psl89* × *psl270*	Wild-type	Non-allelic
*psl117* × *psl270*	Wild-type	Non-allelic

## Supplementary Materials

Supplementary materials can be found at www.mdpi.com/1422-0067/19/1/140/s1.

## References

[1] Sakuraba Y., Han S., Yang H., Piao W., Paek N. (2016). Mutation of *Rice Early Flowering3.1* (*OsELF3.1*) delays leaf senescence in rice. Plant Mol. Biol..

[2] Liang C., Chu C. (2015). Towards understanding abscisic acid-mediated leaf senescence. Sci. China Life Sci..

[3] Morita R., Sato Y., Yu M., Nishimura M., Kusaba M. (2009). Defect in non-yellow coloring 3, an alpha/beta hydrolase-fold family protein, causes a stay-green phenotype during leaf senescence in rice. Plant J..

[4] Liang C., Wang Y., Zhu Y., Tang J., Hu B., Liu L., Ou S., Wu H., Sun X., Chu J. (2014). OsNAP connects abscisic acid and leaf senescence by fine-tuning abscisic acid biosynthesis and directly targeting senescence-associated genes in rice. Proc. Natl. Acad. Sci. USA.

[5] Zheng X., Fan S., Wei H., Tao C., Ma Q., Zhang S., Li H., Pang C., Yu S. (2017). iTRAQ-based quantitative proteomic analysis reveals cold responsive proteins involved in leaf senescence in upland cotton (*Gossypium hirsutum* L.). Int. J. Mol. Sci..

[6] Li F., Hu G., Fu Y., Si H., Bai X., Sun Z. (2005). Genetic analysis and high-resolution mapping of a premature senescence gene *Pse(t)* in rice (*Oryza sativa* L.). Genome.

[7] Huang Q., Shi Y., Zhang X., Song L., Feng B., Wang H., Xu X., Li X., Guo D., Wu J. (2016). Single base substitution in *OsCDC48* is responsible for premature senescence and death phenotype in rice. J. Integr. Plant Biol..

[8] Okada K., Katoh S. (1998). Two long-term effects of light that control the stability of proteins related to photosynthesis during senescence of rice leaves. Plant Cell Physiol..

[9] Jiao B., Wang J., Zhu X., Zeng L., Li Q., He Z. (2012). A novel protein RLS1 with NB-ARM domains is involved in chloroplast degradation during leaf senescence in rice. Mol. Plant.

[10] Kusaba M., Ito H., Morita R., Iida S., Sato Y., Fujimoto M., Kawasaki S., Tanaka R., Hirochika H., Nishimura M. (2007). Rice NON-YELLOW COLORING1 is involved in light-harvesting complex II and grana degradation during leaf senescence. Plant Cell.

[11] Strecker V., Mai S., Muster B., Beneke S., Bürkle A., Bereiter-Hahn J., Jendrach M. (2010). Aging of different avian cultured cells: Lack of ROS-induced damage and quality control mechanisms. Mech. Ageing Dev..

[12] Zhou Q., Yu Q., Wang Z., Pan Y., Lv W., Zhu L., Chen R., He G. (2013). Knockdown of *GDCH* gene reveals reactive oxygen species-induced leaf senescence in rice. Plant Cell Environ..

[13] Li L., Huang Q., Zhang S., Zhao S. (2013). Effect of enhanced UV-B radiation and low-energy N⁺ ion beam radiation on the response of photosynthesis, antioxidant enzymes, and lipid peroxidation in rice (*Oryza sativa*) seedlings. Appl. Biochem. Biotechnol..

[14] Liebsch D., Keech O. (2016). Dark-induced leaf senescence: New insights into a complex light-dependent regulatory pathway. New Phytol..

[15] Mao C., Lu S., Lv B., Zhang B., Shen J., He J., Luo L., Xi D., Chen X., Ming F. (2017). A rice NAC transcription factor promotes leaf senescence via ABA biosynthesis. Plant Physiol..

[16] Hirayama T., Shinozaki K. (2007). Perception and transduction of abscisic acid signals: Keys to the function of the versatile plant hormone ABA. Trends Plant Sci..

[17] Hung K., Kao C. (2004). Hydrogen peroxide is necessary for abscisic acid-induced senescence of rice leaves. J. Plant Physiol..

[18] Liu X., Li Z., Jiang Z., Zhao Y., Peng J., Jin J., Guo H., Luo J. (2011). LSD: A leaf senescence database. Nucl. Acid. Res..

[19] Li Z., Zhao Y., Liu X., Peng J., Guo H., Luo J. (2014). LSD 2.0: An update of the leaf senescence database. Nucl. Acid. Res..

[20] Wu H., Wang B., Chen Y., Liu Y., Chen L. (2013). Characterization and fine mapping of the rice premature senescence mutant *ospse1*. Theor. Appl. Genet..

[21] Kusaba M., Tanaka A., Tanaka R. (2013). Stay-green plants: What do they tell us about the molecular mechanism of leaf senescence. Photosynth. Res..

[22] Jiang H., Li M., Liang N., Yan H., Wei Y., Xu X., Liu J., Xu Z., Chen F., Wu G. (2007). Molecular cloning and function analysis of the *stay green* gene in rice. Plant J..

[23] Sato Y., Morita R., Katsuma S., Nishimura M., Tanaka A., Kusaba M. (2009). Two short-chain dehydrogenase/reductases, NON-YELLOW COLORING 1 and NYC1-LIKE, are required for chlorophyll *b* and light-harvesting complex II degradation during senescence in rice. Plant J..

[24] Rao Y., Yang Y., Xu J., Li X., Leng Y., Dai L., Huang L., Shao G., Ren D., Hu J. (2015). EARLY SENESCENCE_1_ encodes a SCAR-LIKE PROTEIN_2_ that affects water loss in rice. Plant Physiol..

[25] Wu L., Ren D., Hu S., Li G., Dong G., Jiang L., Hu X., Ye W., Cui Y., Zhu L. (2016). Down-regulation of a nicotinate phosphoribosyl transferase gene, *OsNaPRT_1_*, leads to withered leaf tips. Plant Physiol..

[26] Lin A., Wang Y., Tang J., Xue P., Li C., Liu L., Hu B., Yang F., Loake G.J., Chu C. (2012). Nitric oxide and protein *S*-nitrosylation are integral to hydrogen peroxide-induced leaf cell death in rice. Plant Physiol..

[27] Liu L., Sun G., Ren X., Li C., Sun D. (2015). Identification of QTL underlying physiological and morphological traits of flag leaf in barley. BMC Genet..

[28] Aquino-Bolaños E., Mercado-Silva E. (2004). Effects of polyphenol oxidase and peroxidase activity, phenolics and lignin content on the browning of cut jicama. Postharvest Biol. Technol..

[29] Kim H., Ryu H., Hong S., Woo H., Lim P., Lee I., Sheen J., Nam H.G., Hwang I. (2006). Cytokinin-mediated control of leaf longevity by AHK3 through phosphorylation of ARR2 in *Arabidopsis*. Proc. Natl. Acad. Sci. USA.

[30] Kong Z., Li M., Yang W., Xu W., Xue Y. (2006). A novel nuclear-localized CCCH-type zinc finger protein, OsDOS, is involved in delaying leaf senescence in rice. Plant Physiol..

[31] Lim P., Kim H., Nam H. (2007). Leaf senescence. Annu. Rev. Plant Biol..

[32] Su Y., Hu S., Zhang B., Ye W., Niu Y., Guo L., Qian Q. (2017). Characterization and fine mapping of a new early leaf senescence mutant *es3(t)* in rice. Plant Growth Regul..

[33] Lee R., Wang C., Huang L., Chen S. (2001). Leaf senescence in rice plants: Cloning and characterization of senescence up-regulated genes. J. Exp. Bot..

[34] Tang Y., Li M., Chen Y., Wu P., Wu G., Jiang H. (2011). Knockdown of *OsPAO* and *OsRCCR1* cause different plant death phenotypes in rice. J. Plant Physiol..

[35] Lv X., Shi Y., Xu X., Wei Y., Wang H., Zhang X., Wu J. (2015). *Oryza sativa* chloroplast signal recognition particle 43 (OscpSRP43) is required for chloroplast development and photosynthesis. PLoS ONE.

[36] Song Q., Wang Y., Qu M., Ort D., Zhu X. (2017). The impact of modifying photosystem antenna size on canopy photosynthetic efficiency-Development of a new canopy photosynthesis model scaling from metabolism to canopy level processes. Plant Cell Environ..

[37] Pruzinská A., Anders I., Aubry S., Schenk N., Tapernoux-Luthi E., Muller T., Kräutler B., Hörtensteiner S. (2007). In vivo participation of red chlorophyll catabolite reductase in chlorophyll breakdown. Plant Cell.

[38] Shimoda Y., Ito H., Tanaka A. (2016). Arabidopsis *STAY-GREEN*, Mendel’s green cotyledon gene, encodes magnesium-dechelatase. Plant Cell.

[39] Schelbert S., Aubry S., Burla B., Agne B., Kessler F., Krupinska K., Hörtensteiner S. (2009). Pheophytin pheophorbide hydrolase (pheophytinase) is involved in chlorophyll breakdown during leaf senescence in *Arabidopsis*. Plant Cell.

[40] Pruzinská A., Tanner G., Anders I., Roca M., Hörtensteiner S. (2003). Chlorophyll breakdown: Pheophorbide a oxygenase is a Rieske-type iron-sulfur protein, encoded by the accelerated *cell death 1* gene. Proc. Natl. Acad. Sci. USA.

[41] Horie Y., Ito H., Kusaba M., Tanaka R., Tanaka A. (2009). Participation of chlorophyll *b* reductase in the initial step of the degradation of light-harvesting chlorophyll *a/b*-protein complexes in *Arabidopsis*. J. Biol. Chem..

[42] Zhang M., Yuan B., Leng P. (2009). The role of ABA in triggering ethylene biosynthesis and ripening of tomato fruit. J. Exp. Bot..

[43] Zhu G., Ye N., Zhang J. (2009). Glucose-induced delay of seed germination in rice is mediated by the suppression of ABA catabolism rather than an enhancement of ABA biosynthesis. Plant Cell Physiol..

[44] Saika H., Okamoto M., Miyoshi K., Kushiro T., Shinoda S., Nakazono M. (2007). Ethylene promotes submergence-induced expression of *OsABA8ox1*, a gene that encodes ABA 8′-hydroxylase in rice. Plant Cell Physiol..

[45] Gill S., Tuteja N. (2010). Reactive oxygen species and antioxidant machinery in abiotic stress tolerance in crop plants. Plant Physiol. Biochem..

[46] Foyer C., Noctor G. (2003). Redox sensing and signaling associated with reactive oxygen in chloroplasts, peroxisomes and mitochondria. Physiol. Plant.

[47] Apel K., Hirt H. (2004). Reactive oxygen species: Metabolism, oxidative stress, and signal transduction. Annu. Rev. Plant Biol..

[48] Maurino V., Peterhansel C. (2010). Photorespiration: Current status and approaches for metabolic engineering. Curr. Opin. Plant Biol..

[49] Orf I., Timm S., Bauwe H., Fernie A., Hagemann M., Kopka J., Nikoloski Z. (2016). Can cyanobacteria serve as a model of plant photorespiration?—A comparative meta-analysis of metabolite profiles. J. Exp. Bot..

[50] Prochazkova D., Sairam R., Srivastava G., Singh D. (2001). Oxidative stress and antioxidant activity as the basis of senescence in maize leaves. Plant Sci..

[51] Alscher R., Erturk N., Heath L. (2002). Role of superoxide dismutases (SODs) in controlling oxidative stress in plants. J. Exp. Bot..

[52] Zhang Y., Ding S., Lu Q., Yang Z., Wen X., Zhang L., Lu C. (2011). Characterization of photosystem II in transgenic tobacco plants with decreased iron superoxide dismutase. Biochim. Biophys. Acta.

[53] Yin Z., Chen J., Zeng L., Goh M., Leung H., Khush G., Wang G. (2000). Characterizing rice lesion mimic mutants and identifying a mutant with broad-spectrum resistance to rice blast and bacterial blight. Mol. Plant Microbe Interact..

[54] Wellburn A. (1994). The spectral determination of chlorophylls *a* and *b*, as well as total carotenoids, using various solvents with spectrophotometers of different resolution. J. Plant Physiol..

[55] Huang L., Sun Q., Qin F., Li C., Zhao Y., Zhou D. (2007). Down-regulation of a *SILENT INFORMATION REGULATOR_2_*-related histone deacetylase gene, *OsSRT_1_*, induces DNA fragmentation and cell death in rice. Plant Physiol..

