# Supplementary material for: Identification and Comparative Analysis of Premature Senescence Leaf Mutants in Rice (Oryza sativa L.)
Source: Int J Mol Sci. 2018 Jan 3;19(1):140. doi: 10.3390/ijms19010140 (PMC5796089; doi:10.3390/ijms19010140)
Supplement: Supplementary file 1 [file ijms-19-00140-s001.pdf]

Table S1. Primers used in this study for qRT-PCR.

Primers	Forward primers (5'-3')	Reverse primers (5'-3')
Ubiquitin	CCCTCCACCTCGTCCTCAG	AGATAACAACGGAAGCATAAAAGTC
SGR	AGGGGTGGTACAACAAGCTG	GCTCCTTGCGGAAGATGTAG
RCCR1	CGCATTTCCTCATGGAATTT	CTTCTCACGCTGTTTGTCCA
Osh36	AACGCATTTGTGGTTGGCTC	TCAACTTTGGCCGGTGTCTT
OsI57	ACCCTAAAGTAAATGAAGTC	CCTGCTCTTGTCTTGTTA
OsNCED1	ACCATGAAGTCCATGAGGCT	TCTCGTAGTCTTGGTCTTGG
OsNCED3	CAAGTTCGAGTACGGCGAGG	GACAGGATGTAGCCGTCGTC
OsNCED4	TCGGGAGGTACGACTTCCAT	TTGAGGTACGGCTTGGACAC
OsZEP	GGATGCCATTGAGTTTGGTT	TGGCTGACTGAAGTCTCTCG
OsABA8ox1	CAAGCCCAACACGTTCATGC	TGTACTTGGTTGCGAGGTGG
OsABA8ox2	CTACTGCTGATGGTGGCTGA	CCCATGGCCTTTGCTTTAT
OsABA8ox3	AGTACAGCCCATTCCCTGTG	ACGCCTAATCAAACCATTGC
CAO	TTGGCACAAATGGAGACCC	GCTGCACTGGACCAGACAC
rbcS	CCGTGAGAACCACAGATCCC	ACGTTGTCGAAGCCGATGAT
rbcL	ATCGTGCTCGCGGTATCTTT	ACCAGGTGCATTACCCCAAG
HEMA1	ACACGCCATCTGTTTGAGGT	CAAGCCTCCACTGTTTTGCC
psbA	TGTAGCTGGTGTATTCGGCG	ATAACCATGAGCGGCCACAA
porA	ATCACCAAGGGCTACGTCTC	GAGTTGTTGTTCCAGCTCCA
NPH1a	CACTTGCAACCAATGCGTGA	ATCCGGGAGTTCCTTTGCAG
CHLI	CGGAGTAACCTTGGTGCTGT	CTTGGCAGCCCTGTTAGTCA
psbS1	CTGAGCCGAAGCCAAAGTTC	ATCCCCGTCTCCAGGTTCAG
CHLH	TGACTCAGACCCGACAAAGC	TCCCCTCGTACCACTTAGGG
CHLD	GGAAAGAGAGGGCATTAG	CAATACGATCAAGTAAGTGTT
cab2R	GTTCTCCATGTTCGGCTTCT	GACGAAGTTGGTGGCGTAG

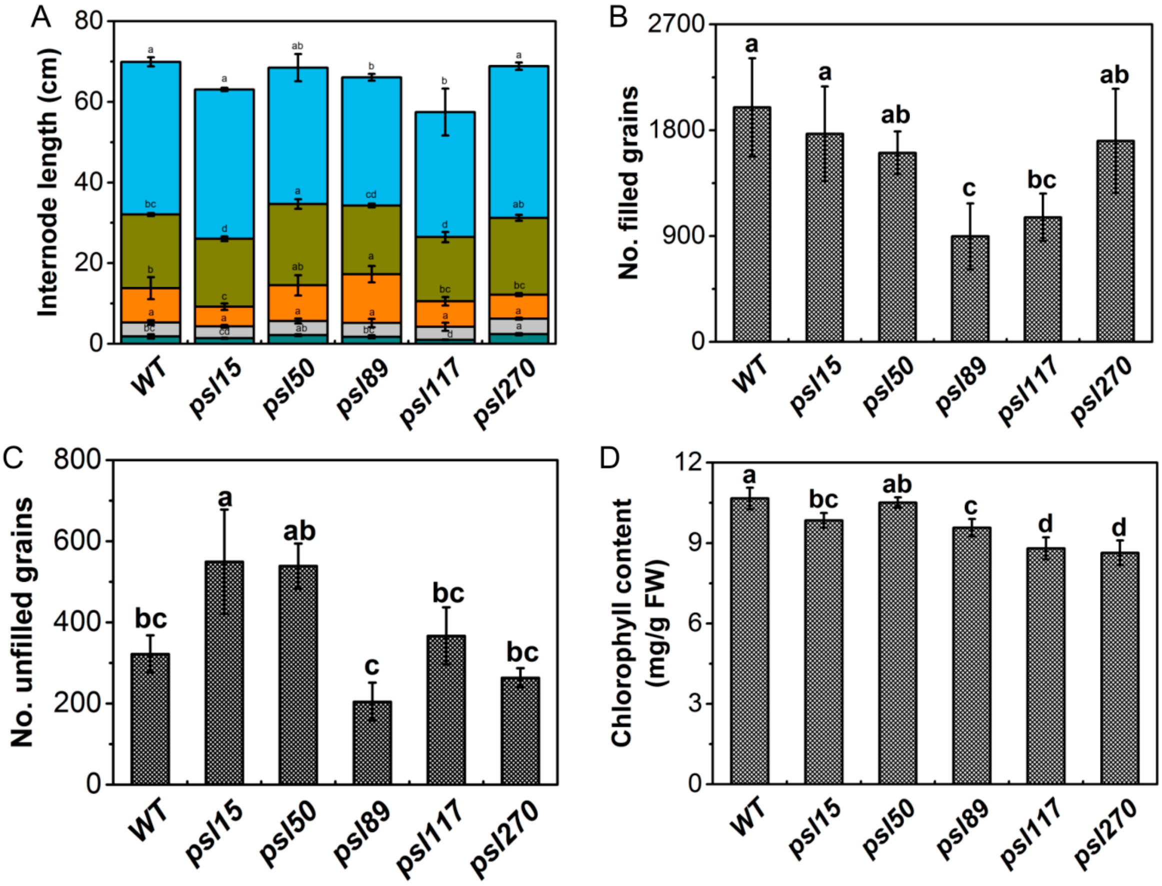

Figure S1. Agronomic traits and chlorophyll contents in WT and mutants. (**A**) internode length of WT and mutants; Columns from bottom to top represent internode Ⅰ, internode Ⅱ, internodeⅢ, internodeⅣ and internodeⅤ, respectively. (**B**) number of filled grain in WT and mutants. (**C**) number of unfilled grain in WT and mutants. (**D**) chlorophyll contents of WT and mutants at the seedling stage. Error bars indicate ±SD (n=3). Different letters above the columns indicate a statistical difference at *P*≤0.05 by Duncan's test.

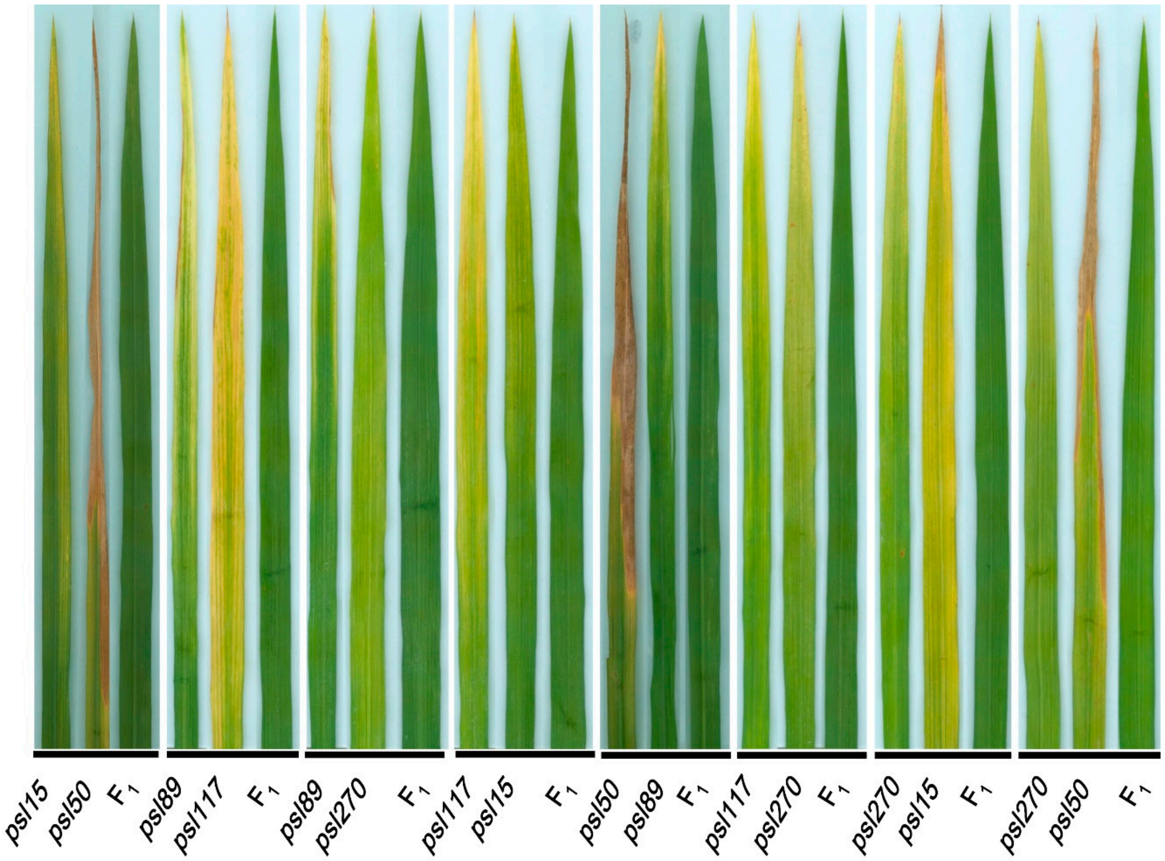

Figure S2. Bottom second leaf phenotypes of parental mutant lines and F_1 plants at the tillering stage.
